# Biaxial tensile behavior of stainless steel 316L manufactured by selective laser melting

**DOI:** 10.1038/s41598-023-49482-7

**Published:** 2023-12-11

**Authors:** Hao Wang, Xiaoyong Shu, Jianping Zhao, I. V. Alexandrov

**Affiliations:** 1 School of Mechanical and Power Engineering, Nanjing Tech University, Nanjing, 211816 People’s Republic of China; 2 Institute of Reliability Centered Manufacturing (IRcM), Nanjing, 211816 People’s Republic of China; 3 Department of Materials Science and Physics of Metals, Ufa University of Science and Technology, Ufa, Russia 450008

**Keywords:** Materials science, Mechanical properties, Metals and alloys

## Abstract

In this study, miniaturized cruciform biaxial tensile specimens were optimized by finite element simulation software Ansys to vary five geometric parameters. The optimized specimens were utilized to characterize the biaxial tensile properties of 316L stainless steel fabricated through selective laser melting (SLM), with the two loading directions being vertical (X) and parallel (Y) to the building direction. It was discovered that at load ratios of 4:2 and 2:4, the yield strengths along X and Y orientations reached their respective maxima. By comparing the experimentally obtained yield loci against predictions by theoretical criteria including Mises, Hill48 and Hosford, it was found that the Hill48 anisotropic criterion corresponded most closely with the experimental results, while the other two criteria exhibited considerably larger deviations. Therefore, Hill48 was concluded to most accurately describe the yielding behaviors of SLM 316L under complex loading conditions.

## Introduction

Selective laser melting (SLM) is a powder bed additive manufacturing technology belonging to the category of powder bed fusion. It works by utilizing a high-power laser to selectively scan and melt the surface of metallic powder spread into a thin layer. This process is repeated layer-by-layer until the complete part is formed. SLM is capable of processing many metallic materials with high precision, directly fabricating complex components without the need for molds^1^. In recent years, SLM has seen rapid development and been widely applied in aerospace^2^, medical^3^, and automotive manufacturing. Compared with conventional methods, SLM technology can significantly reduce material waste, integrate design and manufacturing, enabling lightweight and customized fabrication of components.

316L stainless steel is one of the most widely processed metals by SLM, owing to its high corrosion resistance and excellent combination of strength and ductility after post-processing^4,5^. Due to the excellent mechanical properties, corrosion resistance and biocompatibility of 316L stainless steel, SLM 316L can be used in medical device applications and orthopedic implants^6,7^. Due to the design freedom provided by SLM and the excellent wear and corrosion resistance of 316L, SLM 316L is often used in the manufacture of furnace fixtures, heat exchange piping, turbine blades in jet engines, etc^8^.

However, differences between SLM and conventional manufacturing result in unique microstructures and mechanical anisotropy in SLM-processed 316L parts^9,10^. In the horizontal direction parallel to the build layers, ultimate tensile strength (UTS) and elongation of SLM 316L have been reported to be up to 10% and 60% lower respectively than the vertical direction normal to the layers^9,11,12^. This anisotropy is mainly attributed to the layer banding effects arising from localized remelting during SLM^4,13,14^. Lack-of-fusion defects generated between layers also impart negative effects^15,16^. In addition, rapid solidification and steep thermal gradients in SLM lead to fine cellular dendritic grains and elemental segregation along the build direction, which yield inferior tensile properties horizontally compared to vertically^17–20^. Porosity defects and rough surfaces associated with partially fused powder particles also degrade the horizontal tensile behavior^21–23^.

In summary, the intrinsic SLM process-structure–property relationships result in anisotropic tensile performance for 316L stainless steel. Further efforts are required to fundamentally understand the structure–property correlations, which will provide insights to guide SLM process optimization for balanced mechanical properties^24^. Therefore, investigating the biaxial tensile properties of 316L fabricated by SLM is crucial.

Biaxial tensile testing of metallic materials provides critical insights into their forming limit diagrams (FLDs) and plastic deformation behaviors under complex strain paths^25–27^. This is essential for predicting material formability and guiding the design of manufacturing processes for metallic parts^28^. The cruciform specimen is one of the most commonly adopted sample geometries for biaxial tensile experiments due to its structural symmetry and relatively uniform stress state in the center gauge area^29,30^. The dimensions of a cruciform specimen need to be carefully designed to achieve a balanced biaxial stress state during testing^31–33^. Common design factors include the sample length, width, thickness, fillet radius, and length of gauge area. Proper dimension ratios have been analytically derived and experimentally validated to minimize undesirable stress concentrations for various materials ^31,34–36^. However, optimized geometries often have large volumes, which increases material waste. Therefore, this study utilizes finite element software Ansys to simulate biaxial tension experiments for designing miniaturized cruciform specimens and optimizing their geometric parameters.

Accurately predicting the yielding behaviors of metallic materials is essential for simulating their plastic deformation in manufacturing processes^37,38^. The von Mises, Hill 1948 (Hill48) and Hosford yield criteria are among the most established anisotropic yield functions suitable for various metals^39–43^. The von Mises criterion assumes that yielding occurs when the von Mises stress reaches a critical value, applicable for isotropic metals like mild steel^44^. The Hill48 criterion introduces anisotropy by using different yield stresses along three material directions, providing good accuracy for cubic metals^37,45^. The Hosford criterion generalizes the isotropic von Mises model with a tunable exponent parameter. The exponent value can be fitted to experimental data to capture anisotropic yielding. Compared to Hill48, Hosford yield surfaces have smoother corners better matching measured shapes. Two general yield criteria, isotropic Mises and anisotropic Hill48, are included in commercial nonlinear finite element software ABAQUS. The accuracy of the yield criterion can be verified by the results of biaxial tensile test.

In this study, miniaturized cruciform biaxial tensile specimens was designed and optimized by the finite element software Ansys. The biaxial mechanical behavior of SLM 316L was studied by the biaxial tensile test with the optimized miniaturized cruciform specimens, and the stress–strain curves under different load ratios were obtained. The yield loci of SLM 316L under biaxial stress is obtained by calculation. The yield loci obtained by experiments were compared with those calculated by Mises, Hill48 and Hosford yield criteria to verify the accuracy of different yield criteria. This can provide a reference for the numerical simulation to predict the performance of parts.

## Specimens and methods

### Design optimization of miniaturized cruciform specimens

**Figure 1:**
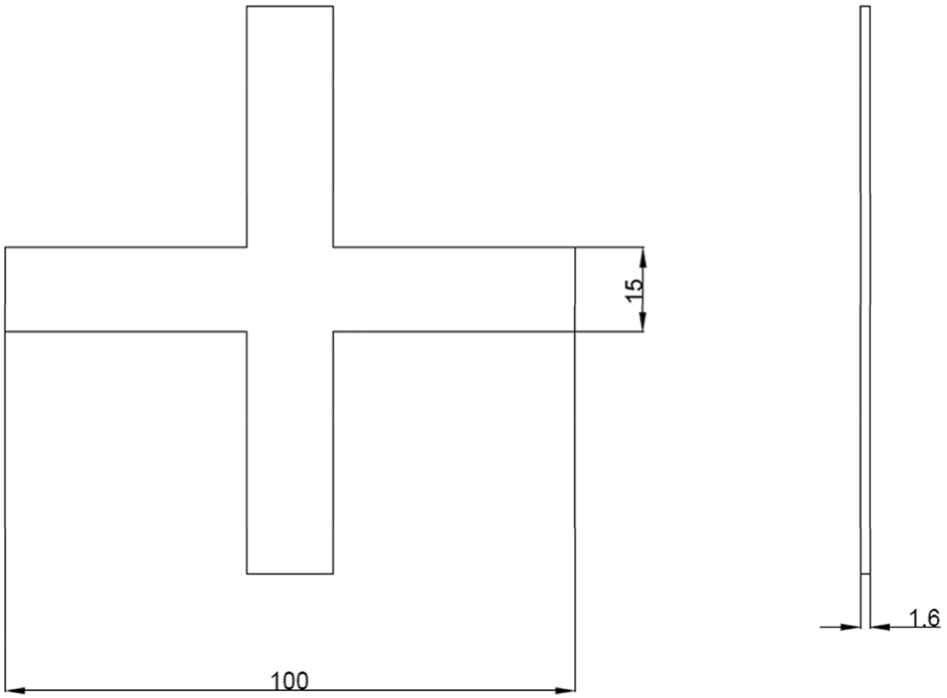
Basic dimensions of cruciform specimens.

**Figure 2:**
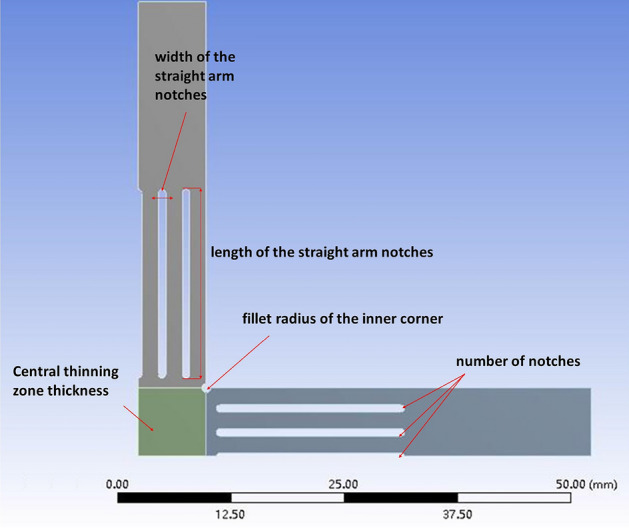
1/4 Model of cruciform specimen.

To conduct biaxial tensile tests, cruciform specimens were designed using finite element analysis software ANSYS. Biaxial tensile simulations were performed to obtain the stress states of the specimens, based on which the geometries were optimized. Tensile tests employed cruciform specimens with overall dimensions shown in Fig. 1. Five geometric factors of the specimens were optimized, including thickness of the center gage section, width of the straight arm notches, length of the straight arm notches, number of notches, and fillet radius of the inner corner. The levels of each factor are listed in Table 1. Through iterative simulation and optimization, a cruciform specimen design inducing balanced biaxial stresses was obtained. The adopted specimen geometry and dimensions enabled reliable characterization of the plastic deformation behavior through subsequent biaxial tensile experiments. A ${L}_{15}({4}^{5})$ orthogonal array was implemented for the orthogonal experimental design. Owing to the cruciform symmetry, only a quarter fraction was modeled for finite element analysis, as illustrated in Fig. 2 showing the FE model of the specimen.

**Table 1:** Levels of geometric shape factors for cruciform specimens.

	Thickness of the center gage section (mm)	Width of the straight arm notches (mm)	Length of the straight arm notches (mm)	Number of notches	Fillet radius of the inner corner (mm)
1	0.8	0.2	5	1	0.25
2	0.6	0.4	7.5	3	0.5
3	0.4	0.6	10	5	0.75
4	0.2	0.8	12.5	7	1

### Materials and additive manufacturing

**Figure 3:**
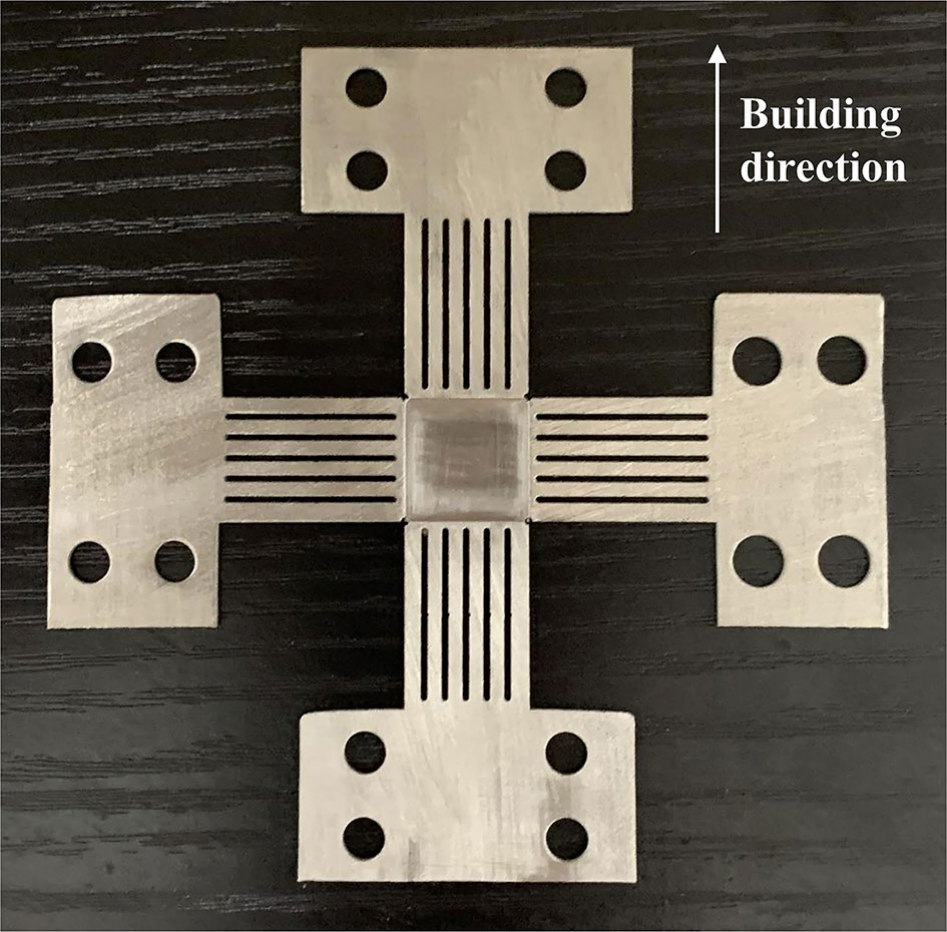
316L cruciform specimens fabricated by SLM.

The SLM process utilized a commercial gas-atomized 316L powder with particle size distribution shown in Table 2 and nominal composition listed in Table 3. Printing occurred under argon atmosphere to prevent oxidation. The adopted processing parameters are provided in Table 4. A reciprocating scanning strategy with 67° rotation between layers was implemented. The directly fabricated cruciform specimen by SLM is depicted in Fig. 3.

**Table 2:** The particle size distribution of powder.

Cumulative distribution (%)	Particle size (μm)
D10	20.22
D50	32.34
D90	51.84

D10, D50 and D90 refer to the particle size corresponding to the accumulative distribution of 10, 50 and 90%, respectively.

**Table 3:** Chemical composition of 316L stainless steel powder.

Element	Weight%
Fe	Balance
C	0.012
Si	0.690
Mn	1.260
P	0.010
S	0.007
Cr	16.470
Ni	12.720
Mo	2.440
O	0.062

**Table 4:** The processing parameters of SLM.

Laser power (W)	400
Scanning speed (mm/s)	1300
Hatch distance (mm)	0.11
Powder layer thickness (mm)	0.06

### Biaxial tension testing

**Figure 4:**
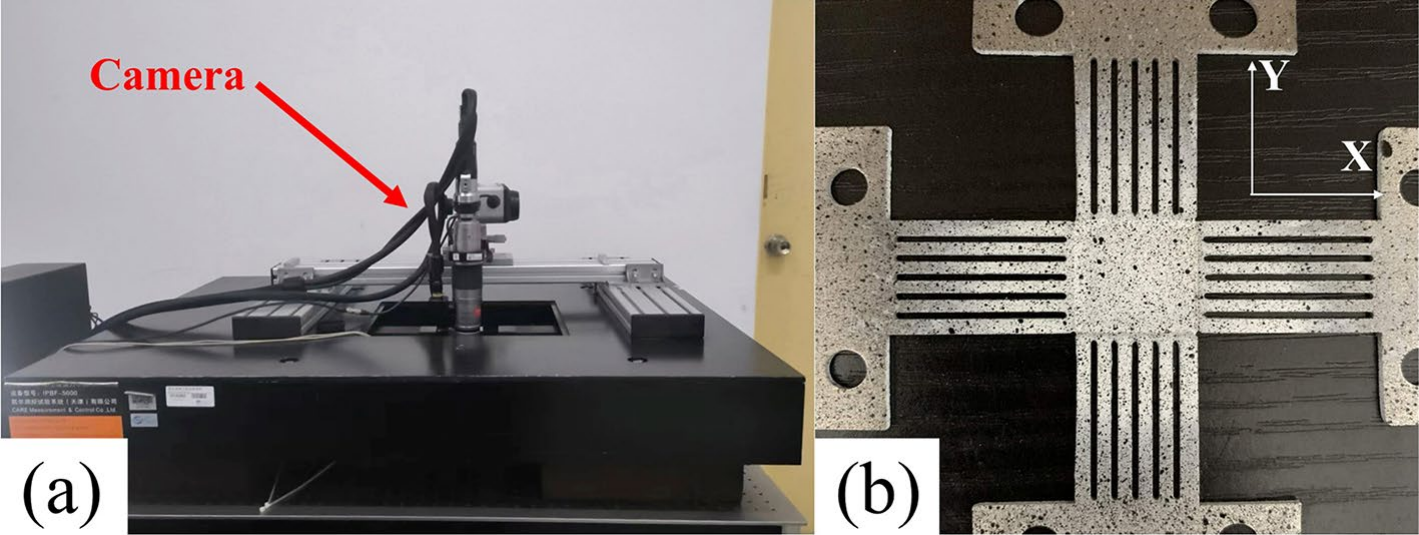
(**a**) DIC and biaxial tensile testing machine, (**b**) speckle pattern.

In situ biaxial tensile tests of SLM 316L cruciform specimens were performed using an IPBF-5000 system (CARE Measurement and Control Co., Tianjin, China). Full-field surface strain measurements were obtained through non-contact digital image correlation (DIC). Speckle patterns were applied on the gage sections (Fig. 4b). A FUJIFILM HF50SA-1 camera (FUJIFILM Holdings Corp., Tokyo, Japan) at 5 megapixel resolution and 9.7° angle captured speckle variations (Fig. 4a). Gaussian prefiltering and bicubic spline interpolation during post-processing reduced displacement measurement errors. The color scale depicts the DIC-determined minimum to maximum strains.

Load-controlled mode with fixed proportional ${F}_{x}:{F}_{y}$ tension loads was implemented, where X denotes vertical to the build direction, and Y parallel. Specific ${F}_{x}:{F}_{y}$ ratios were $4:0, 4:1, 4:2, 4:3, 4:4, 3:4, 2:4, 1:4, 0:4$. Uniaxial tensile test values defined the 4:0 and 0:4 limits^46^.

### Ethical approval

This study did not involve human or animal subjects, and thus, no ethical approval was required. The study protocol adhered to the guidelines established by the journal.

## Results and discussion

### Design optimization of miniaturized cruciform specimens

**Figure 5:**
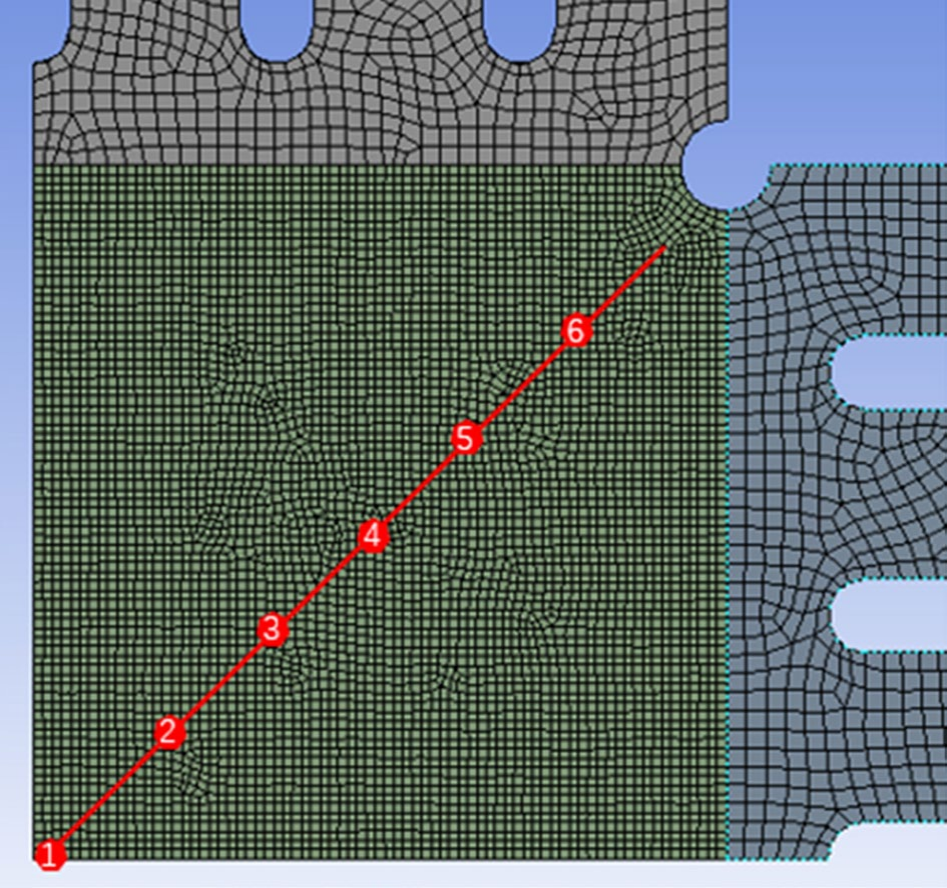
The schematic of the reference nodes.

This study optimized the cruciform specimen geometry focusing on achieving uniform stress distribution in the center gage section. The stress uniformity $\gamma$ of the center gage was defined as:

1 $$ \gamma = \frac{1}{m}\mathop \sum \limits_{i = 1}^{m} \left( {\frac{{\sigma_{mises}^{i} }}{{\sigma_{mises}^{centre} }}} \right)^{2} $$

where m is the number of selected reference points (m = 6, Fig. 5), and ${\sigma }_{mises}^{i}$ is the von Mises stress at each reference node.

Figure 5 illustrates the schematic of the reference nodes.

Finite element models of cruciform specimens with dimensional variations per the factor levels in Table 1 and L15 orthogonal array were generated. Biaxial tensile simulations provided the stress states during loading. The stress uniformity $\gamma$ was calculated using Eq. (1) based on the nodal stresses. Tables 5 and 6 list the computed $\gamma$ values for node 1 stresses of 200 and 500 MPa, respectively. (Factors A, B, C, D, E in the table correspond to the thickness of the center gage section, width of the straight arm notches, length of the straight arm notches, number of notches and fillet radius of the inner corner respectively).

**Table 5:** Orthogonal array and results (${\sigma }_{mises}^{1}$ = 200 MPa).

Experiment number	Factor					Uniformity ($\gamma$)	Test scheme
	A	B	C	D	E		
1	1	1	1	1	1	1.050	${A}_{1}{B}_{1}{C}_{1}{D}_{1}{E}_{1}$
2	1	2	2	2	2	1.054	${A}_{1}{B}_{2}{C}_{2}{D}_{2}{E}_{2}$
3	1	3	3	3	3	1.066	${A}_{1}{B}_{3}{C}_{3}{D}_{3}{E}_{3}$
4	1	4	4	4	4	1.088	${A}_{1}{B}_{4}{C}_{4}{D}_{4}{E}_{4}$
5	2	1	2	3	4	1.075	${A}_{2}{B}_{1}{C}_{2}{D}_{3}{E}_{4}$
6	2	2	1	4	3	1.066	${A}_{2}{B}_{2}{C}_{1}{D}_{4}{E}_{3}$
7	2	3	4	1	2	1.065	${A}_{2}{B}_{3}{C}_{4}{D}_{1}{E}_{2}$
8	2	4	3	2	1	1.048	${A}_{2}{B}_{4}{C}_{3}{D}_{2}{E}_{1}$
9	3	1	3	4	2	1.066	${A}_{3}{B}_{1}{C}_{3}{D}_{4}{E}_{2}$
10	3	2	1	3	1	1.063	${A}_{3}{B}_{2}{C}_{1}{D}_{3}{E}_{1}$
11	3	3	4	2	4	1.092	${A}_{3}{B}_{3}{C}_{4}{D}_{2}{E}_{4}$
12	3	4	2	1	3	1.085	${A}_{3}{B}_{4}{C}_{2}{D}_{1}{E}_{3}$
13	4	1	4	2	3	1.099	${A}_{4}{B}_{1}{C}_{4}{D}_{2}{E}_{3}$
14	4	2	3	1	4	1.104	${A}_{4}{B}_{2}{C}_{3}{D}_{1}{E}_{4}$
15	4	3	2	4	1	1.082	${A}_{4}{B}_{3}{C}_{2}{D}_{4}{E}_{1}$
16	4	4	1	3	2	1.092	${A}_{4}{B}_{4}{C}_{1}{D}_{3}{E}_{2}$

**Table 6:** Orthogonal array and results (${\sigma }_{mises}^{1}$ = 500 MPa).

Experiment number	Factor					Uniformity ($\gamma$)	Test scheme
	A	B	C	D	E		
1	1	1	1	1	1	1.017	${A}_{1}{B}_{1}{C}_{1}{D}_{1}{E}_{1}$
2	1	2	2	2	2	1.018	${A}_{1}{B}_{2}{C}_{2}{D}_{2}{E}_{2}$
3	1	3	3	3	3	1.020	${A}_{1}{B}_{3}{C}_{3}{D}_{3}{E}_{3}$
4	1	4	4	4	4	1.022	${A}_{1}{B}_{4}{C}_{4}{D}_{4}{E}_{4}$
5	2	1	2	3	4	1.020	${A}_{2}{B}_{1}{C}_{2}{D}_{3}{E}_{4}$
6	2	2	1	4	3	1.019	${A}_{2}{B}_{2}{C}_{1}{D}_{4}{E}_{3}$
7	2	3	4	1	2	1.018	${A}_{2}{B}_{3}{C}_{4}{D}_{1}{E}_{2}$
8	2	4	3	2	1	1.017	${A}_{2}{B}_{4}{C}_{3}{D}_{2}{E}_{1}$
9	3	1	3	4	2	1.018	${A}_{3}{B}_{1}{C}_{3}{D}_{4}{E}_{2}$
10	3	2	1	3	1	1.015	${A}_{3}{B}_{2}{C}_{1}{D}_{3}{E}_{1}$
11	3	3	4	2	4	1.019	${A}_{3}{B}_{3}{C}_{4}{D}_{2}{E}_{4}$
12	3	4	2	1	3	1.018	${A}_{3}{B}_{4}{C}_{2}{D}_{1}{E}_{3}$
13	4	1	4	2	3	1.020	${A}_{4}{B}_{1}{C}_{4}{D}_{2}{E}_{3}$
14	4	2	3	1	4	1.019	${A}_{4}{B}_{2}{C}_{3}{D}_{1}{E}_{4}$
15	4	3	2	4	1	1.017	${A}_{4}{B}_{3}{C}_{2}{D}_{4}{E}_{1}$
16	4	4	1	3	2	1.017	${A}_{4}{B}_{4}{C}_{1}{D}_{3}{E}_{2}$

**Figure 6:**
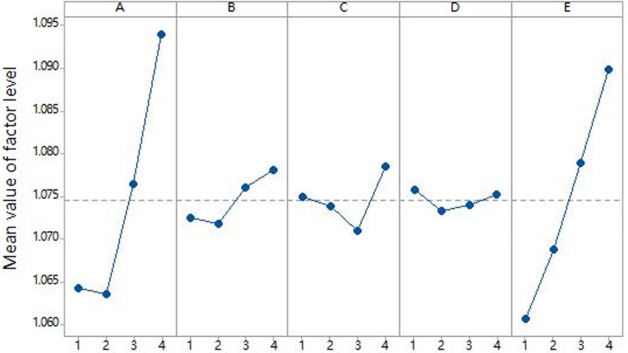
Influence of factor levels on uniformity(200 MPa).

**Figure 7:**
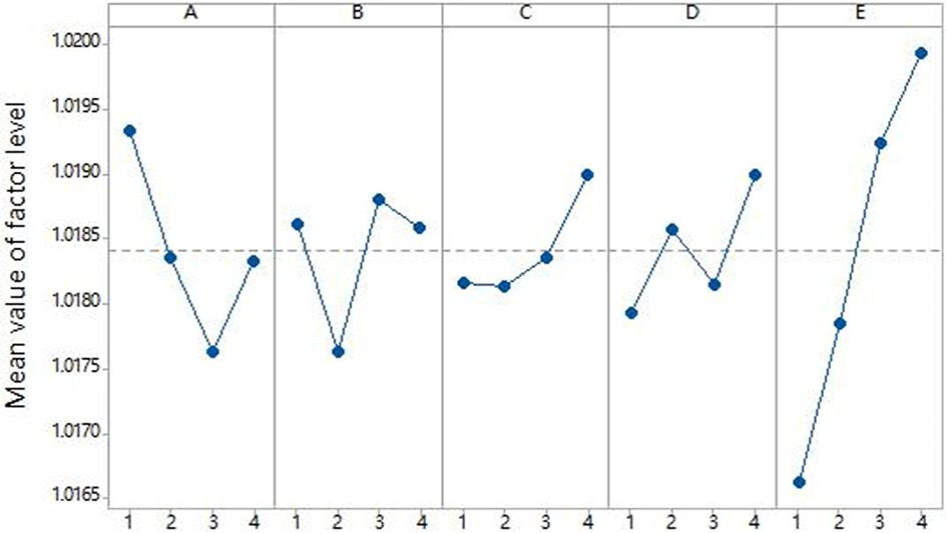
Influence of factor levels on uniformity(500 MPa).

The influence of each factor on $\gamma$ was determined using the Statistica software based on the computed results in Tables 5 and 6, as depicted in Figs. 6 and 7.

Figure 6 indicates geometry ${A}_{2}{B}_{2}{C}_{3}{D}_{2}{E}_{1}$ conferring optimal $\gamma$ under 200 MPa, while Fig. 7 shows geometry ${A}_{3}{B}_{2}{C}_{2}{D}_{1}{E}_{1}$ being optimal for 500 MPa. Although one notch $({D}_{1})$ minimally influenced $\gamma$ at 500 MPa, it exceeded the average effect at 200 MPa. Similarly, three notches $({D}_{2})$ also surpassed the mean impact on $\gamma$ at 500 MPa. In contrast, five notches $({D}_{3})$ exhibited relatively low effects at both stresses. Hence, five notches $({D}_{3})$ were chosen for the notch number. Considering diminished formability with excessively small fillet radius, 0.25 mm ${(E}_{1})$ was replaced with 0.5 mm ${(E}_{2})$. Likewise, the notch width was increased from 0.4 mm ${(B}_{2})$ to 0.8 mm ${(B}_{4})$. Integrating these factors, geometry ${A}_{3}{B}_{4}{C}_{3}{D}_{3}{E}_{2}$ was selected as the optimum cruciform design, with final dimensions listed in Table 7.

**Table 7:** Optimal geometry of cruciform specimens.

	The thickness of the center gage section (mm)	Width of the straight arm notches (mm)	Length of the straight arm notches (mm)	The number of notches	Fillet radius of the inner corner (mm)
Optimal geometry	0.4	0.8	10	5	0.5

### True stress-true strain curves for different loading ratios

To obtain the true stress-true strain curves under nine loading ratios, the area of the mid-plane cross-section in the gage region was defined as S, calculated by:

2 $$S=w\cdot t=\frac{{w}_{0}\cdot {t}_{0}\cdot {l}_{0}}{l}=\frac{{w}_{0}\cdot {t}_{0}}{1+\varepsilon }$$

where ${l}_{0}$, ${w}_{0}$, ${t}_{0}$ are the initial length, width and thickness of the gage section. $l$, $w$, $t$ represent the instantaneous counterparts, and $\varepsilon$ is the strain along the measurement direction perpendicular to the cross-sectional area S. The true stress σ can then be expressed as:

3 $$\sigma =\frac{F}{S}$$

**Figure 8:**
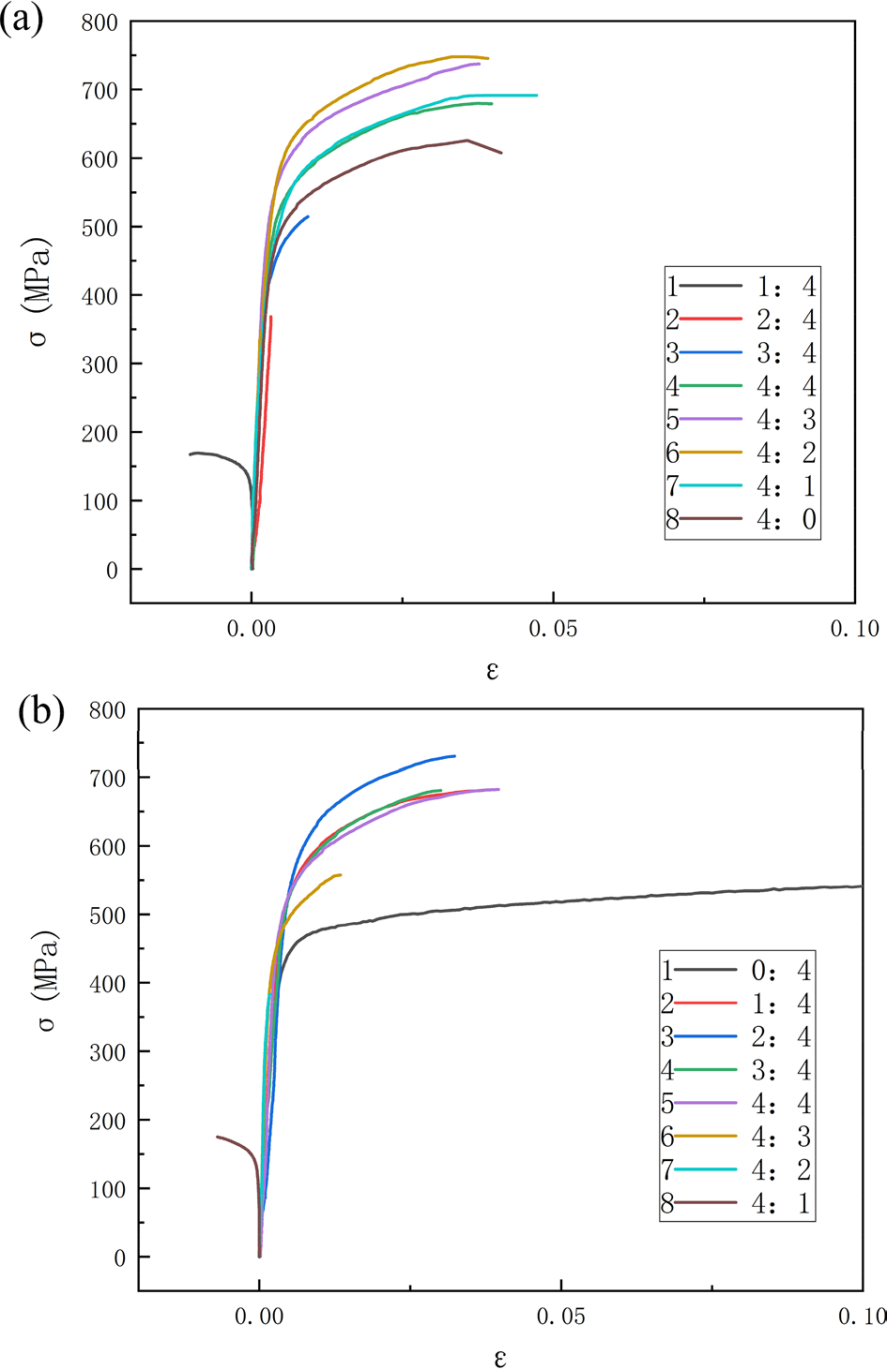
True stress-true strain curve of SLM 316L for different loading ratios, (**a**) X direction, (**b**) Y direction.

**Figure 9:**
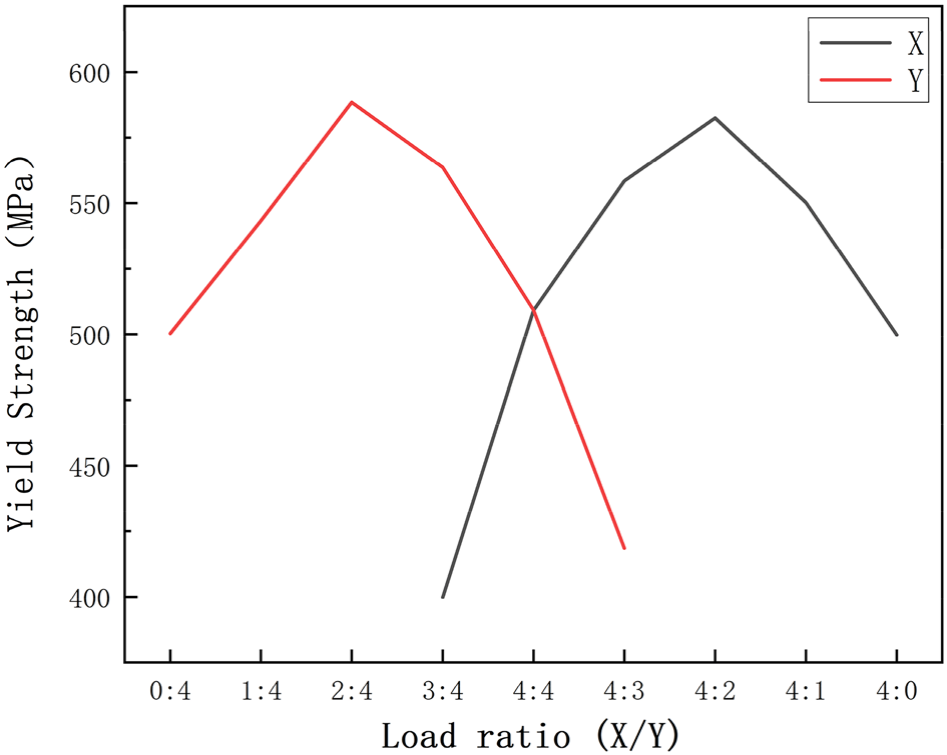
Evolution of yield strength with loading ratios.

Figure 8 presents the true stress-true strain curves of SLM 316L stainless steel under nine biaxial tension loading ratios. The flow behavior varied with the stress state, and the strain hardening exponent increased as the load ratio shifted from uniaxial toward balanced biaxial tension. Table 8 lists the yield strengths along the two directions under various loading ratios. Figure 9 presents the evolution of yield strengths along the two directions with varying loading ratios. The peaks were attained at 4:2 and 2:4 ratios for the respective orientations, differing from conventionally forged 316L that typically peaks at equivalent ratios^47^.

**Table 8:** Biaxial tensile tests data at various load ratios.

Testing direction	Load ratio (X/Y)	Yield strength at X (MPa)	Yield strength at Y (MPa)
Uniaxial	0:4		500.3
Biaxial	1:4		543.2
Biaxial	2:4		588.4
Biaxial	3:4	399.8	563.6
Biaxial	4:4	509.1	509.2
Biaxial	4:3	558.4	418.5
Biaxial	4:2	582.5	
Biaxial	4:1	550.2	
Uniaxial	4:0	499.7

### Experimental yield locus

To determine the yield points for the tensile tests, true plastic strains ${\varepsilon }_{x}^{p}$ and ${\varepsilon }_{y}^{p}$ along the X and Y orientations were calculated using Eq. (4).

4 $${\varepsilon }_{x}^{p}={\varepsilon }_{x}-\frac{{\sigma }_{x}}{{C}_{x}};{\varepsilon }_{y}^{p}={\varepsilon }_{y}-\frac{{\sigma }_{y}}{{C}_{y}}$$

where ${C}_{x}$ and ${C}_{y}$ are the slopes of the elastic portions of curves ${\varepsilon }_{x}-{\sigma }_{x}$ and ${\varepsilon }_{y}-{\sigma }_{y}$ measured in MPa from the biaxial tensile tests. ${\varepsilon }_{x}$ and ${\varepsilon }_{y}$ represent the true strains along the X and Y orientations, respectively.

**Figure 10:**
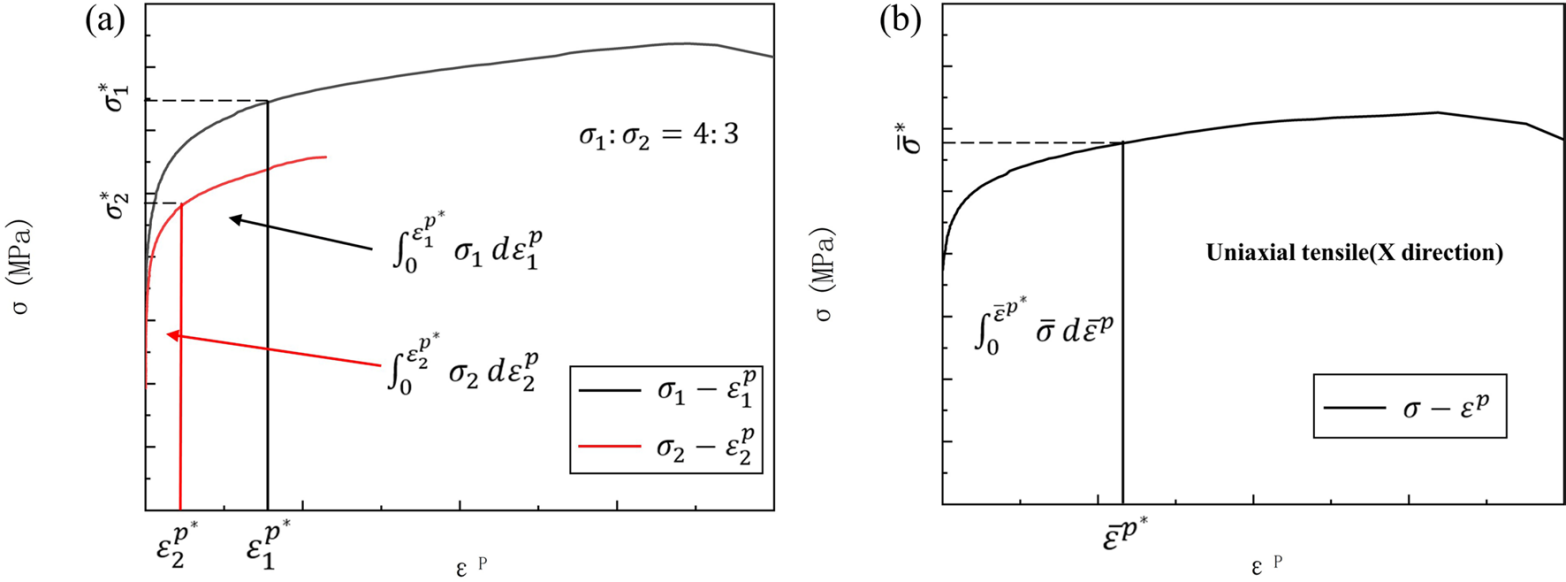
Determination procedures of plastic work contours. (**a**) Biaxial tensile condition; (**b**) uniaxial tensile condition.

For simplicity, plastic work contours are often considered equivalent to experimentally measured yield point trajectories^48^. Uniaxial tensile tests on SLM 316L vertical to the build direction using ASTM E8 specimens provided uniaxial true stresses ${\sigma }_{0}^{p}$ corresponding to plastic strains ${\varepsilon }_{0}^{p}$ of 0.002, 0.006 and 0.01. The plastic work W was measured per unit of plastic strain ${\varepsilon }_{0}^{p}$. In biaxial tensile tests with fixed stress ratios, the sum of plastic work along both orientations was obtained. Equivalent yield loci were identified when the unit plastic works W were equal under different stress states. For instance, as depicted in Fig. 10, point $\left({\sigma }_{1}^{p*},{\sigma }_{2}^{p*}\right)$ represents a given biaxial tension stress state^49^. Point $\left({\tilde{\sigma }}^{*},{\overline{\varepsilon }}^{p*}\right)$ refers to the uniaxial stress–strain curve vertical to the building direction, satisfying:

5 $${\int }_{0}^{{\sigma }_{1}^{p*}}{\sigma }_{1}d{\varepsilon }_{1}^{p}+{\int }_{0}^{{\sigma }_{2}^{p*}}{\sigma }_{2}d{\varepsilon }_{2}^{p}={\int }_{0}^{{\overline{\varepsilon }}^{p*}}\tilde{\sigma }d{\overline{\varepsilon }}^{p}$$

**Figure 11:**
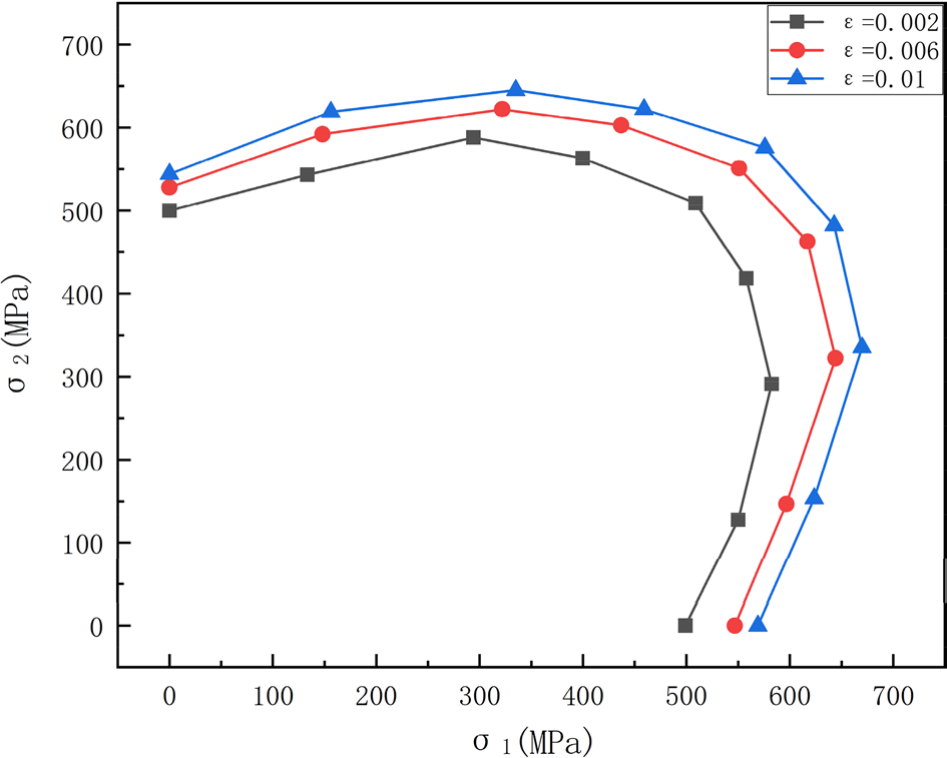
The experimental yield locus of SLM 316L at equivalent plastic strain 0.2, 0.6 and 1%.

Notably, the integration in Eq. (5) was performed using discrete numerical integration, specifically integrating discrete trapezoidal areas encompassed by the data points and horizontal axis. This method yielded yield loci at three equivalent plastic strain levels for SLM 316L, as depicted in Fig. 11.

The yield contours demonstrate similar evolving trends in shape and size with increasing plastic deformation. Per the convexity principle, the plastic work contours expand outwards. Owing to strain hardening, the contours intensify from center to periphery at a given plastic strain increment. Notably, anisotropic mechanical properties induced asymmetry in the yield locus shapes along the balanced biaxial tension path, deviating from classic isotropic predictions. This signifies that deformation history and direction dependency in the SLM-processed 316L stainless steel influence yielding even at relatively small strains. The expanded yield area indicates enhanced formability, but the asymmetric distortions suggest potentially complex yielding characteristics under multi-axial loading.

### Comparison and analysis of experimental and theoretical locus

**Figure 12:**
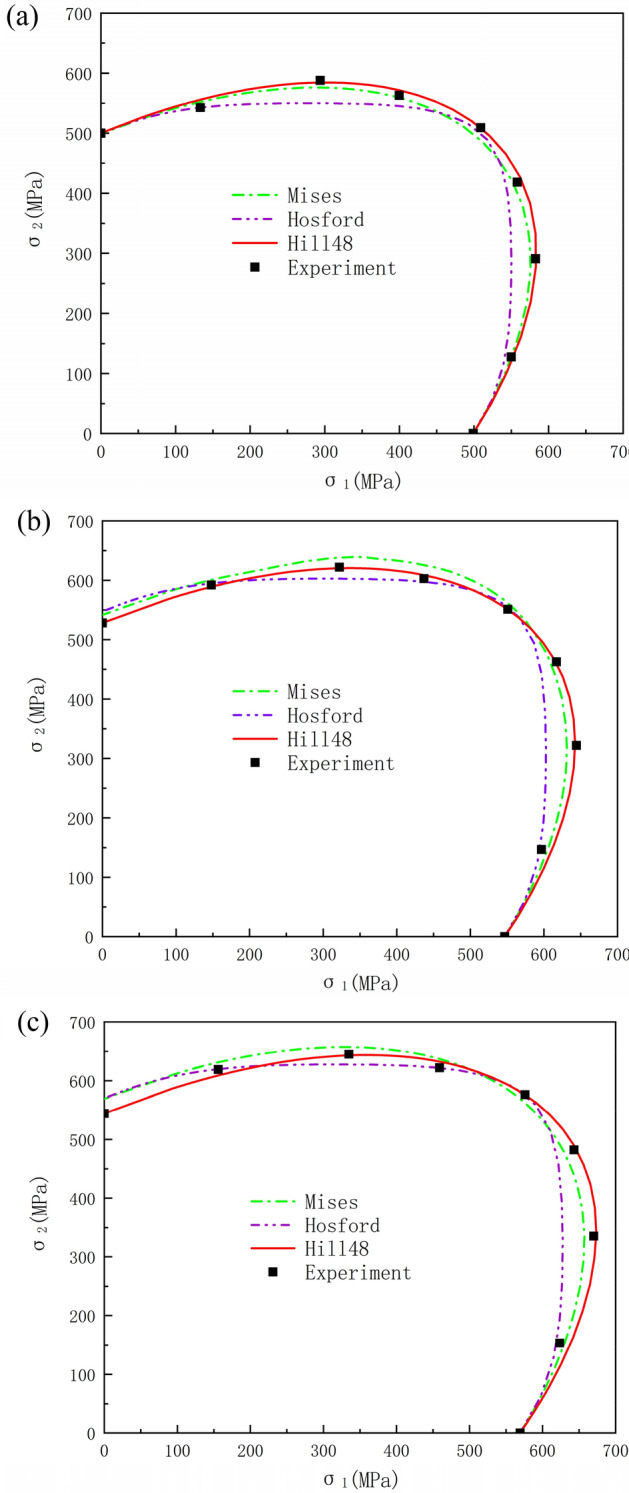
Comparison between experimental and calculated yield loci. (**a**) ${\varepsilon }_{0}^{p}=0.002$, (**b**) ${\varepsilon }_{0}^{p}=0.006$, (**c**) ${\varepsilon }_{0}^{p}=0.01$.

**Figure 13:**
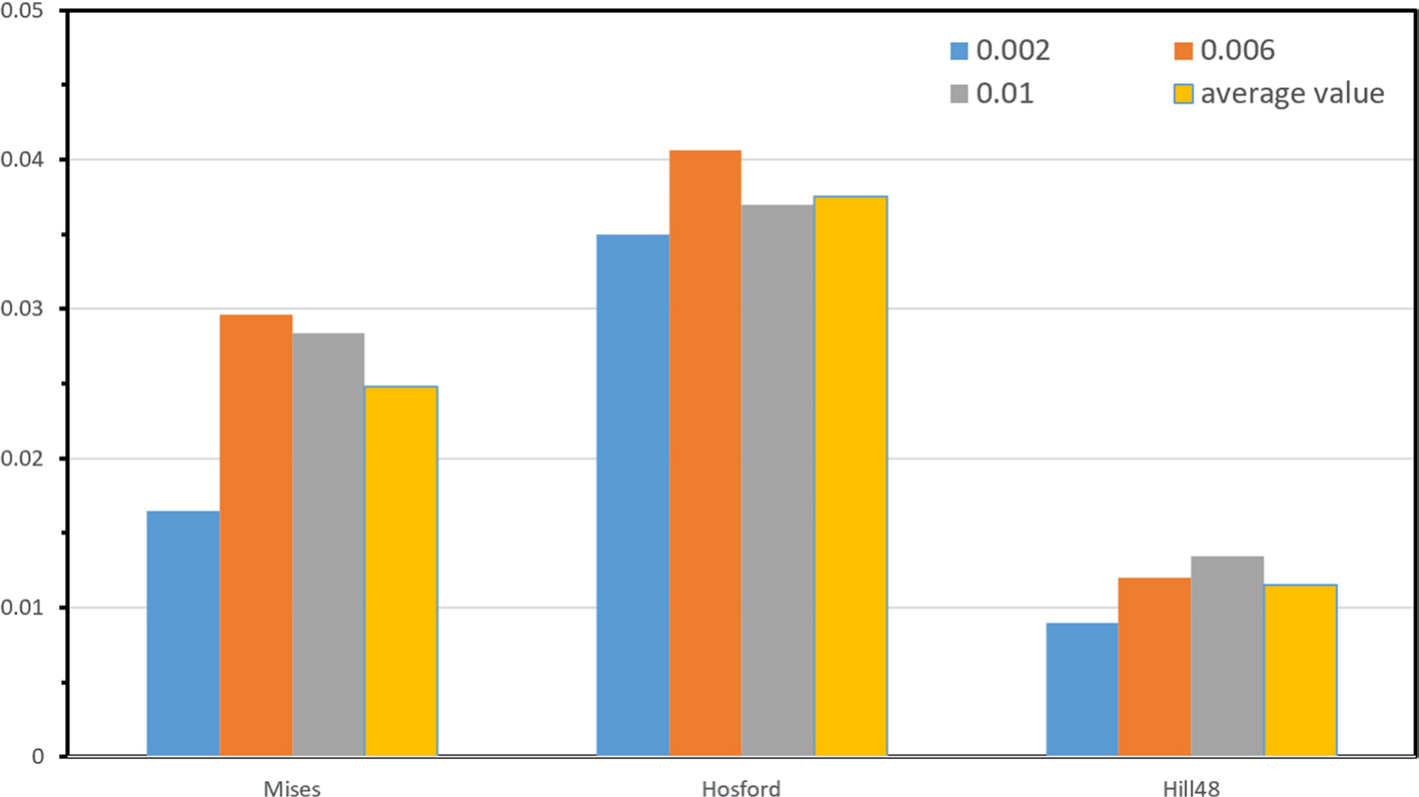
The mean errors between experimental and calculated values.

This section compares the experimentally obtained yield loci against predictions by theoretical yield criteria (Mises, Hill48, Hosford). The calculation of the parameters in theoretical yield criterion is adequately elaborated in Refs.^40,43^. To quantify the correspondence between the calculated and measured yield points, the mean error $\delta$ was defined as an accuracy metric:

6 $$\delta =\frac{1}{n}\sum_{i=1}^{n}\frac{{d}_{i}}{\sqrt{{\left({\sigma }_{1}^{i}\right)}^{2}+{\left({\sigma }_{2}^{i}\right)}^{2}}}$$

where $\left({\sigma }_{1}^{i},{\sigma }_{2}^{i}\right)$ denotes the experimental yield point coordinates, ${d}_{i}$ is the normal distance from the point to the calculated yield contour, and n is the number of experimental points. Figure 12 presents the measured yield loci versus those predicted by theoretical criteria. Figure 13 shows the mean errors between experimental and calculated values.

As shown in Fig. 12, the Hosford yield criteria deviated considerably from experiments under 4:2 and 2:4 loading ratios, with significant inaccuracies in balanced biaxial tension prediction. The Hosford yield criteria, grounded in crystalline plasticity principles, precludes shear stress components in its formulation. However, SLM 316L stainless steel possesses intricate grain morphological distributions, which the limited anisotropy parameters in the Hosford yield criteria struggle to fully delineate. Additionally, the Hosford criterion does not incorporate shear stresses, that notably sway yielding responses of anisotropic alloys under complex loading states. This underpins appreciable deviations between Hosford-predicted and experimentally observed yield loci, especially for biaxial tension conditions, in the SLM 316L alloy. Although the Mises criterion exhibited moderately improved alignment with experimental yield loci, its inherent isotropy prevented capturing the experimentally observed yield contour asymmetry induced by the anisotropic behavior of SLM 316L. Consequently, inadequate conformance persisted between the symmetric Mises predictions and the asymmetric measured yield loci. Comparatively, the Hill48 contour aligned closely with experiments, which Fig. 13 quantifies. The Hosford criterion had the largest mean error versus experiments, while the Hill48 error was markedly lower than the other two models. As a phenomenological criterion on the macroscale, the Hill48 yield criteria can delineate the yielding behavior of materials with intricate grain distributions, like SLM 316L stainless steel, with relatively high accuracy despite only using a limited set of anisotropy parameters. Therefore, Hill48 is more suitable for describing the multi-axis yield behavior of SLM 316L. Moreover, all criteria showed increased deviations at higher versus lower strains per Fig. 13.

## Summary and conclusions

In this work, biaxial tension simulations were conducted via Ansys to optimize the dimensional parameters of miniaturized cruciform specimens. The optimized samples were then fabricated from 316L stainless steel by SLM, and experimentally tested under biaxial loading to characterize the mechanical performance. Their yield loci obtained computationally were benchmarked against loci predicted by theoretical yield criteria. The key conclusions are:

1. The biaxial stress–strain response of SLM 316L stainless steel was loading-ratio-dependent, with the yield strengths along the two orientations reaching respective maxima at 4:2 and 2:4 ratios.
2. Adhering to the concept of plastic work contours, the yield loci of SLM 316L were obtained through computations. It was revealed that with increasing plastic deformation, the yield loci expanded outwards following the convexity principle. Moreover, asymmetry became evident in the calculated yield contours, attributed to the inherent anisotropy of 316L fabricated by selective laser melting.
3. Comparisons between experimentally obtained yield loci and model predictions based on Mises, Hill48 and Hosford criteria reveal the Hill48 anisotropic function as most accurately capturing the measured yielding behavior of SLM 316L under complex loading. The other two criteria deviated considerably from experiments. Thus, the Hill48 anisotropic criterion optimally delineates the anisotropic yield characteristics of additively manufactured 316L stainless steel.

## Data Availability

Data will be made available on request (Hao Wang: wh_njtech@163.com).
